# *Gynura bicolor* aqueous extract attenuated H_2_O_2_ induced injury in PC12 cells

**DOI:** 10.1051/bmdcn/2019090212

**Published:** 2019-05-24

**Authors:** Ya-Chen Yang, Wen-Tzu Wu, Mei-Chin Mong, Zhi-Hong Wang

**Affiliations:** 1 Department of Food Nutrition and Health Biotechnology, Asia University Taichung 413 Taiwan; 2 Department of Medical Research, China Medical University Hospital, China Medical University Taichung 404 Taiwan

**Keywords:** Gynura bicolor, NGF-PC12 cell, Apoptosis, NF-κB, p38

## Abstract

Background: Protective effects of *Gynura bicolor* aqueous extract (GAE) at three concentrations upon nerve growth factor (NGF) differentiated-PC12 cells against H_2_O_2_ induced injury were examined.

Methods: NGF differentiated-PC12 cells were treated with GAE at 0.25%, 0.5% or 1%. 100 μM H_2_O_2_ was used to treat cells with GAE pre-treatments. After incubating at 37 °C for 12 hr, experimental analyses were processed.

Results: H_2_O_2_ exposure decreased cell viability, increased plasma membrane damage, suppressed Bcl-2 mRNA expression and enhanced Bax mRNA expression. GAE pre-treatments reversed these changes. H_2_O_2_ exposure reduced mitochondrial membrane potential, lowered Na^+^-K^+^-ATPase activity, and increased DNA fragmentation and Ca^2+^ release. GAE pre-treatments attenuated these alterations. H_2_O_2_ stimulated the production of reactive oxygen species (ROS), interleukin (IL)-1beta, IL-6 and tumor necrosis factor-alpha, lowered glutathione content, and reduced glutathione peroxidase (GPX) and catalase activities. GAE pretreatments maintained GPX and catalase activities; and concentration-dependently diminished the generation of ROS and inflammatory cytokines. H_2_O_2_ enhanced mRNA expression of nuclear factor kappa (NF-κ) B and p38. GAE pre-treatments decreased mRNA expression of NF-κB and p38. Conclusion: These findings suggested that GAE might be a potent neuronal protective agent.

## 1. Introduction

Oxidative and inflammatory reactions are involved in nigral degeneration and neuronal cell death, which contribute to the pathogenesis of neurological disorders such as Parkinson’s disease (PD) [1]. The over-generated oxidants and inflammatory cytokines including reactive oxygen species (ROS), interleukin (IL)-1β, IL-6 and tumor necrosis factor (TNF)-alpha cause neuronal cells apoptosis, impair brain functions, and deteriorate PD and/or other neurological diseases [2]. In addition, the increased caspase activity, Bax expression, and nuclear transcription factor kappa (NF-κ) B activation due to some stimulants also promote damage, and even death of neuronal cells [3, 4]. Thus, exploring the appropriate natural agent(s) with the capabilities to decrease the production, activity or expression of these above factors might be a good and safe choice in order to enhance the stability of neuronal cells, prevent or attenuate the progression of neurological disorders. PC12 cell line, a rat adrenal gland pheochromocytoma cell line, could become a sympathetic neuronal phenotype through reacting with nerve growth factor (NGF) for differentiation [5]. So far, NGF treated PC12 cells have been considered as sympathetic neurons to investigate the protective effects and action modes of some potent compounds for neuronal cells [6, 7].

*Gynura bicolor* DC. (*G. bicolor*) is a plant food, and available in several Asian countries such as China, Taiwan, Japan and Malaysia. Its leaf part is an edible vegetable. *G. bicolor* has been applied in folk medicine for diabetes treatment in China southern area [8]. Tuekpe *et al*. [9] reported that dietary *G. bicolor* intake promoted urinary potassium excretion, which benefited the management of blood pressure for healthy Japanese women. The study of Teoh *et al*. [10] revealed that component compounds of *G. bicolor* exhibited cytotoxic effects for colon cancer cells. Wu *et al*. [11] reported that *G. bicolor* water or ethanol extract enhanced iron bioavailability in rats. In the study by Chao *et al*. [12], four groups of phytochemicals including flavonoids, phenolic acids, carotenoids and anthocyanins were detected in aqueous extract of *G. bicolor* leaf part, and their content were 1934, 1428, 921 and 2135 mg/100 g dry weight. Furthermore, this aqueous extract displayed anti-oxidative activities for human umbilical vein endothelial cells against high glucose [12]. In addition, our previous animal study found that dietary intake of *G. bicolor* aqueous extract (GAE) markedly attenuated hepatic glycative injury and lipid accumulation in mice with chronic ethanol consumption [13], and the authors indicated that the observed hepatic protective activities from GAE were due to the contribution of its phytochemical component compounds. These previous studies suggest that GAE could offer multiple bioactivities. Therefore, it is hypothesized that GAE might be able to protect neuronal cells.

In order to understand whether GAE could be developed as a neuro-protective agent, our present cell line study was conducted. NGF differentiated-PC12 cells were pre-treated with GAE at three concentrations. Then, hydrogen peroxide was used to induce apoptotic, oxidative and inflammatory stress. The effects of GAE on cell survival, plasma membrane integration, caspases and Na^+^-K^+^-ATPase activities, and mRNA expression of Bcl-2, Bax, NF-κB and p38 were examined. Furthermore, the anti-oxidative and anti-inflammatory activities of GAE against H_2_O_2_ were also evaluated. These results could partially support and explain the possibility of considering GAE as a neuro-protective nutraceutical.

## 2. Materials and methods

### 2.1. Materials

Fresh *G. bicolor* was directly purchased from farms in spring, 2015. 100 gram fresh leaf part was cut into small pieces, and mixed with 250 *ml* double distilled water. After homogenizing in a blender, GAE was collected *via* filtrating through a No. 1 whatman filter paper. GAE was further freeze-dried to fine powder. The content of total phenolic acids and total flavonoids in GAE were in the range of 1428 ± 137 and 1934 ± 108 mg/100 g fine powder [12]. In our present work, the levels of total phenolic acids and total flavonoids were measured in order to standardize the used GAE. NGF was purchased from Sigma Chemical Co. (St. Louis, MO, USA). Antibodies were obtained from Boehringer-Mannheim Co. (Indianapolis, IN, USA). Culture medium, plates and chemicals for cell culture were bought from Difco Laboratory (Detroit, MI, USA).

### 2.2. PC12 cell culture and treatments

PC12 cells cultured in Dulbecco’s modified Eagle’s medium (DMEM) were routinely maintained under 95% air and 5% CO_2_ at 37 °C. PC12 cells were treated by NGF at 50 ng/*ml*, and followed by a 5-day incubation at 37 °C. Medium was refreshed every 72 hr. After washing twice with serum-free DMEM, cells were collected and loaded in 96 well plates. Cell number was adjusted to 10^5^/*ml* by phosphate buffer saline (PBS). GAE was dissolved in DMEM. Two groups of NGF differentiated-PC12 cells were treated with 500 μL DMEM only; they were a normal group and a control group, respectively. Three groups of NGF differentiated-PC12 cells were treated with 500 μL DMEM containing GAE at 0.25%, 0.5% or 1%. After incubation for 48 hr at 37 °C, cell samples were washed twice with serum-free DMEM. Then, those used serum-free DMEM was collected, and the content of phenolic acids, flavonoids, carotenoids or anthocyanins was analyzed according to the methods described in Chao *et al*. [12]. There were no detectable phenolic acids, flavonoids, carotenoids or anthocyanins in the DMEM used for washing. Subsequently, 100 μM H_2_O_2_ was used to treat control group, and three groups of cells with GAE pre-treatments. After incubating at 37 °C for 12 hr, experimental analyses were processed.

### 2.3. Cell survival and plasma membrane damage

Cell survival was measured by 3-(4,5-dimethylthiazol-2-yl)-2,5- diphenyltetrazolium bromide (MTT) assay. In brief, MTT at 0.25 mg/mL was added into cell suspension, and this mixture was incubated at 37 °C for 3 hr. MTT formazan product was quantified by monitoring the absorbance at 570 nm by a Bio-Rad microplate reader (Hercules, CA, USA). Cell viability was presented as a percentage of normal groups. Plasma membrane damage was assayed by determining lactate dehydrogenase (LDH) activity. After centrifugation, 50 μL supernatant was used to measure LDH activity (U/L) by a kit (Sigma Chemical Co., St. Louis, MO, USA) according to manufacturer’s instruction.

### 2.4. Assays for DNA fragmentation and mitochondrial membrane potential (MMP)

DNA fragmentation was determined by a cell death detection ELISA kit (Roche Molecular Biochemicals, Mannheim, Germany) according to manufacturer’s instruction. Cells were suspended in cold lysis buffer for 30 min at 25 °C, and centrifuged for 10 min at 250 xg. Twenty μL supernatant was used to react with 80 μL freshly prepared immunoreagent, and followed by incubating for 2 hr at 25 °C. After washed twice with PBS, substrate was added and followed by incubating for 15 min at room temperature. A microplate reader was applied to monitor the absorbance at 405 nm and 490 nm. DNA fragmentation was shown as an enrichment factor, which means: (absorbance of the sample) / (absorbance of the control groups). MMP was measured by using Rh123, a fluorescent dye. Cell samples were treated with Rh123 at 100 μg/L for 30 min at 37 °C. After washed twice with PBS, the mean fluorescence intensity (MFI) was analyzed by a Beckman-FC500 flow cytometry (Beckman Coulter, Fullerton, CA, USA).

### 2.5. Measurement of caspases and Na^+^-K^+^-ATPase activities

Caspase-3 and caspase-8 activities were quantified by fluorometric kits (Upstate, Lake Placid, NY, USA) according to manufacturer’s instructions. Cells were lysed, and protein concentration was determined by a Pierce assay kit (Rockford, IL, USA). The lysates were reacted with specific substrates, and followed by incubating 60 min at 37 °C. Fluorescence value was recorded by a Hitachi F-4500 fluorophotometer (Tokyo, Japan), in which excitation and emission wavelengths were 400 nm and 505 nm. The variability coefficients of inter-assay and intra-assay were 3.9- 5.6% and 4.3-5.9%, respectively. Caspase-3 or caspase-8 activity was defined as fluorescence unit/mg protein. Na^+^-K^+^-ATPase activity was assayed according to the method of Torlinska and Grochowalska [14] *via* analyzing the released amount of inorganic phosphate (Pi) from ATP. The released Pi was determined by monitoring the absorbance at 640 nm. The value of the treated groups was shown as a percentage of normal groups.

### 2.6. Assay of intracellular Ca^2+^ level

A Ca^2+^-sensitive dye, Fura-2AM, was used to detect the intracellular Ca^2+^ level *via* recording the change in fluorescent intensity [15]. In brief, Fura-2AM at 5 mmol/L was added into cells (105 cells/mL), and stored in dark condition for 30 min at 25 °C. After further incubating 30 min at 37 °C, fluorescence value was recorded by a Shimadzu spectrofluorimeter (Model RF-5000, Kyoto, Japan). The emission wavelength was set at 510 nm, and excitation wavelength was set at 340 and 380 nm. Calcium concentration (nM) was calculated according to the equation: [Ca^2+^] = Kd × [(R–Rmin)/(Rmax–R)] × FD/FS. Kd was 224 nM, R was the ratio of fluorescence values at 340 and 380, Rmax was measured by using triton X-100 to treat cells, Rmin was measured by using ethylene glycol tetraacetic acid to treat cells. FD was the fluorescence value of Ca^2+^-free form, and FS was the fluorescence value of Ca^2+^-bound form at 380 and 340 nm.

### 2.7. Assays for oxidative and inflammatory associated factors

ROS level was determined by 2’,7’-Dichlorofluorescein diacetate (DCFH-DA). In brief, 100 μL cell homogenate was mixed with 100 μL 2 mg/*ml* DCFH-DA. After incubating at 37 °C for 30 min, fluorescence value was recorded by a Gemini XS fluorescence plate reader (Molecular Devices, Sunnyvale, CA, USA). Emission and excitation wavelengths were 525 nm and 488 nm, respectively. Result was expressed as relative fluorescence unit (RFU) per mg protein. The level of glutathione (GSH), and the activity of glutathione peroxidase (GPX) or catalase were measured *via* assay kits purchased from OxisResearch Co. (Portland, OR, USA) according to manufacturer’s instructions. The levels (pg/mg protein) of IL-1beta, IL-6 and TNF-alpha were quantified by cytoscreen assay kits obtained from BioSource International Co. (Camarillo, CA, USA). The detection limit was 5 pg/mg protein.

### 2.8. Real-time polymerase chain reaction (RT-PCR) for mRNA expression

Total mRNA of cells was extracted by reagents obtained from Invitrogen Trizol (Life Technologies, Carlsbad, CA, USA). RNA concentration was quantified by monitoring the absorbance at 260 nm. Subsequently, 5 μg RNA was applied for generating cDNA *via* reverse-transcription procedure. Then, cDNA was further used for PCR process. The primers of target genes were as follow: Bcl-2: forward, 5’-CGT TTG GCA GTG CAA TGG T-3’, reverse, 5’-TTC TTG ATT GAG CGA GCC TT-3’; Bax: forward, 5’-TGG CAG CTG ACA TGT TTT CTG AC-3’, reverse, 5’-TCA CCC AAC CAC CCT GGT CTT-3’; NF-κB, forward, 5’-GAG GTC TCT GGG GGT ACA GTC-3’, reverse, 5’-GGA CAA CGC AGT GGA ATT TTA-3’; p38: forward, 5’-TCC AAG GGC TAC ACC AAA TC-3’, reverse, 5’-TGT TCC AGG TAA GGG TGA GC-3’; glyceraldehyde-3-phosphate dehydrogenase (GAPDH, the housekeeping gene): forward, 5’-AGA GGC AGG GAT GTT CTG-3’, reverse, 5’-GAC TCA TGA CCA CAG TCC ATG C-3’. PCR amplification condition was 3 min denaturation at 95 °C, 10 s annealing at 60 °C and 20 s extension at 72 °C. For Bcl-2, Bax, NF-κB or p38, 35 cycles were performed; for GADPH, 28 cycles were processed. A Sequence Detection System (ABI Prism 7700, Applied Biosystems, Foster City, CA, USA) was used to quantify PCR products.

### 2.9. Statistical analyses

Data were obtained from 7 different preparations, and expressed as mean ± standard deviation (SD) (n = 7). Statistical analyses were processed by using one-way analysis of variance. Dunnett’s *t*-test was applied for *Post-hoc* comparison. *P* value lower than 0.05 was defined as significant.

## 3. Results

### 3.1. GAE alleviated apoptosis and plasma membrane damage

**Fig. 1:**
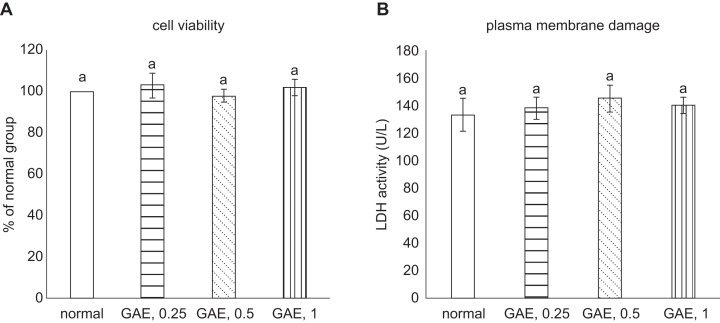
Effects of GAE upon cell viability (a) and plasma membrane damage (b) without H_2_O_2_ treatment. NGF differentiated- PC12 cells were treated with GAE at 0.25%, 0.5% or 1%. Normal group had no GAE. Data are mean ± sd (n = 7). ^a^Values among bars without a common letter differ, *P* < 0.05.

**Fig. 2:**
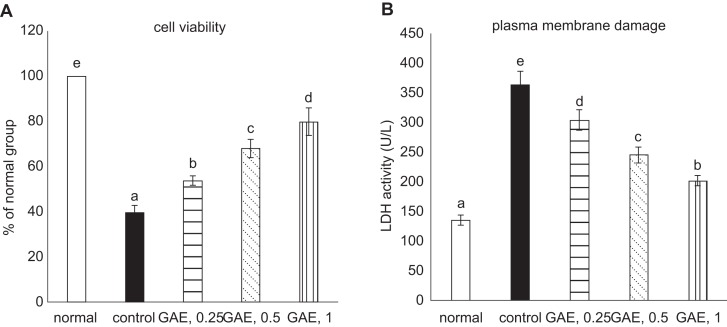
Effects of GAE upon cell viability (a) and plasma membrane damage (b) with H_2_O_2_ treatment. NGF differentiated-PC12 cells were pre-treated with GAE at 0.25%, 0.5% or 1%, and followed by using H_2_O_2_ to induce cell apoptosis. Normal group had no GAE or H_2_O_2_. Control group had no GAE, but with H_2_O_2_. Data are mean ± sd (n = 7). ^a-e^Values among bars without a common letter differ, *P* < 0.05.

**Fig. 3:**
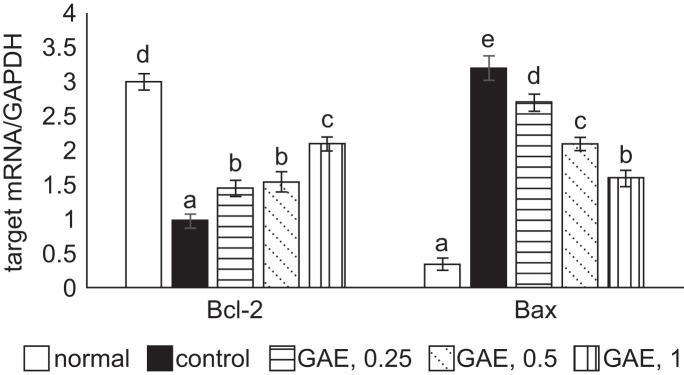
Effects of GAE upon mRNA expression of Bcl-2 and Bax. NGF differentiated-PC12 cells were pre-treated with GAE at 0.25%, 0.5% or 1%, and followed by using H_2_O_2_ to induce cell apoptosis. Normal group had no GAE or H_2_O_2_. Control group had no GAE, but with H_2_O_2_. Data are mean ± sd (n = 7). ^a-e^Values among bars without a common letter differ, *P* < 0.05.

Without H_2_O_2_ stimulation, GAE at test concentrations did not affect viability and plasma membrane stability in NGF differentiated-PC12 cells (Fig. 1a and 1b, *P* < 0.05). As shown in Fig. 2, H_2_O_2_ exposure decreased cell viability (2a) and increased plasma membrane damage (2b), determined by LDH activity, in NGF differentiated-PC12 cells when compared with normal groups (*P* < 0.05). GAE pre-treatments concentration-dependently increased cell viability and diminished LDH activity (*P* < 0.05). H_2_O_2_ reduced Bcl-2 mRNA expression and enhanced Bax mRNA expression in NGF differentiated-PC12 cells (Fig. 3, *P* < 0.05). GAE pre-treatments at test concentrations raised Bcl-2 mRNA expression (*P* < 0.05); and concentration-dependently lowered Bax mRNA expression (*P* < 0.05).

### 3.2. GAE attenuated mitochondrial and DNA injury

**Fig. 4:**
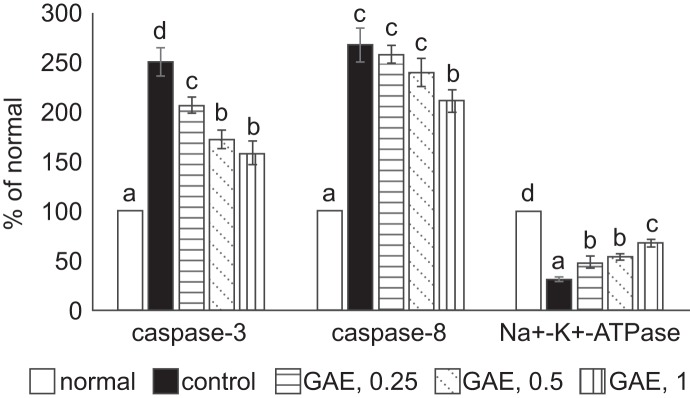
Effects of GAE upon the activity of caspase-3, caspase-8 and Na^+^-K^+^-ATPase. NGF differentiated-PC12 cells were treated with GAE at 0.25%, 0.5% or 1%, and followed by using H_2_O_2_ to induce cell injury. Normal group had no GAE or H_2_O_2_. Control group had no GAE, but with H_2_O_2_. Data are mean ± sd (n = 7). ^a-d^Values among bars without a common letter differ, *P* < 0.05.

As presented in Table 1, H_2_O_2_ exposure reduced MMP, increased DNA fragmentation and Ca^2+^ release in NGF differentiated-PC12 cells (*P* < 0.05). GAE pre-treatments reversed these changes (*P* < 0.05), in which concentration-dependent effects were presented in increasing MMP and reducing DNA fragmentation (*P* < 0.05). H_2_O_2_ exposure enhanced caspase-3 and caspase-8 activities; and lowered Na^+^-K^+^-ATPase activity in NGF differentiated-PC12 cells (Fig. 4, P < 0.05). GAE pre-treatments at test concentrations decreased caspase-3 activity and increased Na^+^-K^+^-ATPase activity (*P* < 0.05). However, GAE pre-treatment only at 1% reduced caspase-8 activity (*P* < 0.05).

**Table 1:** Effects of GAE upon MMP, measured as MFI; DNA fragmentation, measured as enrichment factor; and Ca^2+^ release. NGF differentiated-PC12 cells were pre-treated with GAE at 0.25%, 0.5% or 1%, and followed by using H_2_O_2_ to induce cell injury. Normal group had no GAE or H_2_O_2_. Control group had no GAE, but with H_2_O_2_ . Data are mean ± sd (n = 7). ^a-e^Values in a column without a common letter differ, *P* < 0.05.

	MFI	enrichment factor	[Ca^2+^], nM
Normal	100^e^	1.00^a^	461 ± 49^a^
Control	28 ± 2^a^	2.31 ± 0.12^e^	1618 ± 143^d^
GAE, 0.25	39 ± 4^b^	2.01 ± 0.09^d^	1290 ± 98^c^
GAE, 0.5	54 ± 6^c^	1.69 ± 0.10^c^	1007 ± 56^b^
GAE, 1	68 ± 3^d^	1.4 ± 0.07^b^	927 ± 48^b^

### 3.3. GAE mitigated oxidative and inflammatory stress

**Fig. 5:**
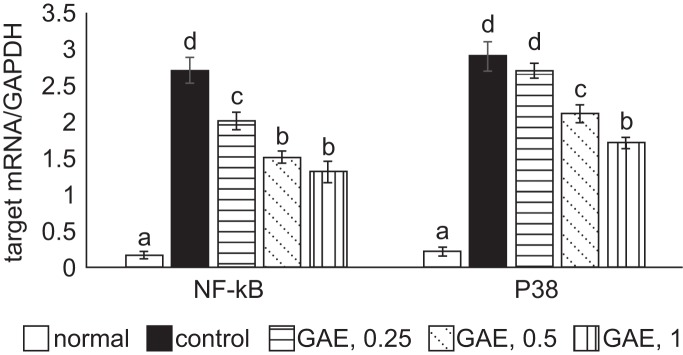
Effects of GAE upon mRNA expression of NF-κB and p38. NGF differentiated-PC12 cells were treated with GAE at 0.25%, 0.5% or 1%, and followed by using H_2_O_2_ to induce cell injury. Normal group had no GAE or H_2_O_2_. Control group had no GAE, but with H_2_O_2_. Data are mean ± sd (n = 7). a-dValues among bars without a common letter differ, *P* < 0.05.

As presented in Table 2, H_2_O_2_ stimulated ROS generation, decreased GSH content, and reduced GPX and catalase activities in NGF differentiated-PC12 cells (*P* < 0.05). GAE pre-treatments reversed GSH content and maintained GPX activity (*P* < 0.05). GAE pre-treatments concentration-dependently lowered ROS level and raised catalase activity (*P* < 0.05). As presented in Table 3, H_2_O_2_ stimulated the release of inflammatory cytokines, IL-1beta, IL-6 and TNF-alpha in NGF differentiated-PC12 cells (*P* < 0.05). GAE pre-treatments decreased the production of these inflammatory cytokines (*P* < 0.05), and concentration-dependent manner was found in lowering TNF-alpha generation (*P* < 0.05). H_2_O_2_ up-regulated mRNA expression of NF-κB and p38 in NGF differentiated-PC12 cells (Fig. 5, *P* < 0.05). GAE pre-treatments at test concentrations down-regulated NF-κB mRNA expression (*P* < 0.05), and GAE pre-treatments at 0.5% and 1% suppressed p38 mRNA expression (*P* < 0.05).

**Table 2:** Effects of GAE upon ROS and GSH levels, and GPX and catalase activities. NGF differentiated-PC12 cells were pre-treated with GAE at 0.25%, 0.5% or 1%, and followed by using H_2_O_2_ to induce cell injury. Normal group had no GAE or H_2_O_2_. Control group had no GAE, but with H_2_O_2_. Data are mean ± sd (n = 7). ^a-e^Values in a column without a common letter differ, *P* < 0.05.

	ROS RFU/mg protein	GSH ng/mg protein	GPX U/mg protein	catalase U/mg protein
Normal	0.14 ± 0.06^a^	90 ± 5^d^	66 ± 4^d^	2.52 ± 0.19^e^
Control	2.36 ± 0.13^e^	39 ± 4^a^	38 ± 2^a^	0.97 ± 0.08^a^
GAE, 0.25	2.00 ± 0.05^d^	49 ± 3^b^	45 ± 3^b^	1.27 ± 0.11^b^
GAE, 0.5	1.58 ± 0.1^c^	62 ± 2^c^	52 ± 4^c^	1.58 ± 0.12^c^
GAE, 1	1.17 ± 0.09^b^	66 ± 3^c^	55 ± 3^c^	1.89 ± 0.07^d^

**Table 3:** Effects of GAE upon level (pg/mg protein) of IL-1beta, IL-6 and TNF-alpha. NGF differentiated-PC12 cells were pre-treated with GAE at 0.25%, 0.5% or 1%, and followed by using H_2_O_2_ to induce cell injury. Normal group had no GAE or H_2_O_2_. Control group had no GAE, but with H_2_O_2_. Data are mean ± sd (n = 7). ^a-e^Values in a column without a common letter differ, *P* < 0.05.

	IL-1beta	IL-6	TNF-alpha
Normal	10 ± 5^a^	9 ± 3^a^	8 ± 4^a^
Control	76 ± 7^d^	79 ± 5^d^	91 ± 6^e^
GAE, 0.25	62 ± 4^c^	60 ± 6^c^	75 ± 4^d^
GAE, 0.5	48 ± 2^b^	46 ± 4^b^	58 ± 3^c^
GAE, 1	45 ± 4^b^	41 ± 3^b^	42 ± 4^b^

## 4. Discussion

The data of our present work revealed that without H_2_O_2_ stimulation, GAE treatments at test concentrations did not affect viability and plasma membrane integrity of NGF differentiated-PC12 cells. These findings implied that GAE might not have adverse impact for neuronal cells. Our previous animal study reported that dietary GAE intake at 0.5% markedly attenuated ethanol-induced hepatic glycative damage and lipid accumulation [13]. Our current cell line study found that GAE at three test concentrations effectively protected NGF differentiated-PC12 cells against subsequent H_2_O_2_ induced apoptotic, oxidative and inflammatory stress. Thus, our previous and present studies supported that GAE might be able to prevent or alleviate chronic diseases *via* its multiple bioactivities.

Bcl-2 is an anti-apoptotic molecule, and Bax is a pro-apoptotic molecule [16]. Both Bcl-2 and Bax are involved in the regulation of neuronal cells survival and/or apoptosis [17]. In our present work, H_2_O_2_ exposure down-regulated Bcl-2 mRNA expression and up-regulated Bax mRNA expression. Consequently, it was reasonable to observe the death of NGF differentiated-PC12 cells. However, the pre-treatments from GAE at test concentrations resulted in greater Bcl-2 mRNA expression and less Bax mRNA expression, which in turn enhanced anti-apoptotic defense and improved cell viability. These results suggest that GAE could mediate Bcl-2/Bax pathway and increase cell survival. On the other hand, H_2_O_2_ exposure impaired plasma membrane integrity and caused DNA fragmentation, which definitely contributed to cell rupture and apoptosis 18, 19]. Our data revealed that GAE pre-treatments markedly overcame the impact from H_2_O_2_ and reversed these alterations. These findings indicated that GAE could benefit DNA stability and maintain the integrity of plasma membranes, which consequently favored cell survival. In addition, GAE pre-treatments diminished H_2_O_2_ induced Ca^2+^ release in NGF differentiated-PC12 cells. The less Ca^2+^ release observed in GAE treated NGF differentiated-PC12 cells could be partially ascribed to the improvement from GAE upon plasma membrane integrity. It is reported that released Ca^2+^ facilitates nerve impulse transmission and stimulates neuronal excitability, which might promote the development and progression of seizure [20, 21]. Our data implied that GAE might decrease neuronal excitability through limiting Ca^2+^ release. Further study regarding the antiseizure effect of GAE is worthy to be investigated.

Collapse of MMP activates apoptotic executors such as caspase-3 and caspase-8 [22]. The raised activity of these two caspases further induced alterations in cellular morphological characteristics and nuclear protein cleavage, and all these events led to cell death [23]. Na^+^-K^+^-ATPase, a transmembrane protein, is in charge of intracellular Na^+^ exchange for extracellular K^+^. The loss of MMP caused the reduction in Na^+^-K^+^-ATPase activity, which subsequently impaired ion homeostasis and promoted apoptotic insult [24]. Unterberg *et al*. [25] indicated that lower Na^+^-K^+^-ATPase activity contributed to neuronal swelling and even brain edema. In our present study, H_2_O_2_ exposure disturbed mitochondrial membrane, which was evidenced by greater caspase-3 and caspase-8 activities, as well as lower Na^+^-K^+^-ATPase activity. However, GAE pre-treatments attenuated mitochondrial membrane injury caused by H_2_O_2_. One possibility was that GAE enhanced the defensive capability of mitochondrial membrane against H_2_O_2_, which consequently diminished the impact from H_2_O_2_ upon caspase-3, caspase-8 and Na^+^-K^+^-ATPase. The other possibility was that GAE directly affected caspases and Na^+^-K^+^- ATPase activities, which finally mitigated apoptotic stress and benefited Na^+^/K^+^ ion homeostasis. These data once again suggest that GAE could maintain mitochondrial membrane stability and alleviated apoptotic stress in H_2_O_2_-treated NGF differentiated- PC12 cells.

It is reported that GAE contained many phytochemicals with anti-oxidative and anti-inflammatory activities such as ferulic acid, chlorogenic acid, quercetin and apigenin [12]. Actually, the anti-oxidative and anti-inflammatory protection of ferulic acid and quercetin for neuronal cells or brain tissue has been reported [26, 27]. Thus, the less production of ROS and inflammatory cytokines, higher GSH content, greater GPX and catalase activities as we observed in GAE treated NGF differentiated-PC12 cells could be ascribed to the presence of phytochemicals in GAE.

We believe that the mitigated oxidative and inflammatory stress also contributed to stabilize DNA and mitochondrial membrane integrity in GAE treated NGF differentiated-PC12 cells, which in turn improved cell survival. As observed by others, the activation of NF-κB and p38 signaling pathways due to H_2_O_2_ stimulation facilitated the generation of oxidants and inflammatory factors such as ROS and TNF-alpha [28, 29]. Our data agreed that H_2_O_2_ was a promotor responsible for neuronal cell oxidative and inflammatory injury. However, our findings indicated that GAE pre-treatments limited the mRNA expression of NF-κB and p38. It is highly possible that the pre-treatments of GAE led to some phytochemical components penetrate plasma membrane of NGF differentiated-PC12 cells, where these component compounds exert their protective actions against subsequent H_2_O_2_ assault. Since these signaling pathways have been suppressed, the lower production of downstream factors such as ROS and inflammatory cytokines could be explained. These finding also suggest that GAE was able to protect NGF differentiated-PC12 cells at molecular levels.

*G. bicolor* is a vegetable. Its aqueous extract is easily prepared and should be safe. Moreover, our previous animal study reported that dietary GAE protected liver against ethanol induced injury [13]. This animal study supported that the active component compounds of GAE could be absorbed, metabolized and exerted its bioactivities. However, it remains unknown that GAE could pass blood brain barrier, and protect brain or neurons. Further animal study is definitely necessary to verify the protective effects of GAE upon brain or neurons. In addition, the phytochemical profile of GAE might not be consistent due to environmental factors such as seasons and planting conditions. Thus, standardization process is very important for the used GAE.

In conclusion, aqueous extract of G. bicolor leaf part enhanced NGF differentiated-PC12 cells survival against H_2_O_2_ through maintaining mitochondrial membrane potential, decreasing oxidative and inflammatory injury, and regulating the mRNA expression of Bcl-2, Bax, NF-κB and p38. These findings suggested that this aqueous extract might possess neuronal protective potent.
